# A Study of Symptomatology of COVID-19 Laboratory-Confirmed Cases at Tertiary Care Center: A Cross-Sectional Study

**DOI:** 10.7759/cureus.22186

**Published:** 2022-02-13

**Authors:** Sandeep Dabhekar, Shrikrishna Basagoudanavar, Vijay Bidkar, Kirankumar Prathipati, Akkilagunta Sujiv, Bharat Sing Rathod, Deepa Gadwal

**Affiliations:** 1 Otolaryngology-Head and Neck Surgery, All India Institute of Medical Sciences, Nagpur, IND; 2 Community Medicine, All India Institute of Medical Sciences, Nagpur, IND; 3 General Medicine, All India Institute of Medical Sciences, Nagpur, IND; 4 Anatomy, Navodaya Medical College, Raichur, IND

**Keywords:** cough, sars-cov-2, central india, symptomatology, covid-19

## Abstract

Background and objectives

People with coronavirus disease 2019 (COVID-19) have had a wide range of symptoms reported such as fever or chills, cough, shortness of breath or difficulty breathing, fatigue, muscle or body aches, headache, a new loss of taste or smell, sore throat, congestion or runny nose, nausea or vomiting and diarrhea. The severity of disease, mortality, symptoms of COVID-19 showed significant variation in different parts of the world. The purpose of this study was to describe epidemiological characteristics and symptoms of confirmed COVID-19 patients and to identify factors associated with the severity of the disease.

Methods

This is a cross-sectional descriptive study conducted on hospitalized COVID-19 patients from May 2020 to July 2020. We obtained data on the demographic characteristics, symptoms, and infection severity for 150 patients by pre-tested semi-structured interview. Information was recorded in a Microsoft Excel sheet and exported to SPSS Statistics (Armonk, NY: IBM Corp.) for analysis.

Results

The median age of the patients was 31.5 years, where 42% of the patients were female; 52.7% of patients were symptomatic while 47.3% of patients were asymptomatic. Common symptoms at the time of admission were fever (40.5%), sore throat (36.7%), cough (32.9%), rhinitis (19.0%), and body ache (13.9%). At least one comorbidity was reported in 20.0% of the patients, with the most common comorbidity being hypertension (14.7%). History of contact with known confirmed cases of COVID-19 within the last 14 days was present in 94% of patients. The presence of any coexisting illness was significantly higher among patients with severe disease than among those with non-severe disease (80% vs. 17.9%, p=0.012).

Conclusions

High proportions of COVID-19 patients were asymptomatic in our study. Fever and cough were the most common symptoms. The presence of any coexisting illness was significantly higher among patients with severe disease than among those with a non-severe disease.

## Introduction

The first human cases of coronavirus disease 2019 (COVID-19), the disease caused by the novel coronavirus, subsequently named severe acute respiratory syndrome coronavirus 2 (SARS-CoV-2) were first reported by officials in Wuhan City, China, in December 2019. From December 30, 2019, through August 31, 2021, over 216 million COVID-19 cases and 4.5 million deaths have been reported globally [1]. Initially, fever, cough, and shortness of breath were considered as primary COVID-19 symptoms. The Centers for Disease Control and Prevention has added six new symptoms of COVID-19 on April 27, 2020, namely chills, repeated shaking with chills, muscle pain, headache, sore throat, and a sudden loss of taste or smell. People with COVID-19 have had a wide range of symptoms reported such as fever or chills, cough, shortness of breath or difficulty breathing, fatigue, muscle or body aches, headache, the new loss of taste or smell, sore throat, congestion, or runny nose, nausea or vomiting, and diarrhea [2].

On reviewing the literature, it has been found that severity of disease, mortality, and symptoms of COVID-19 show significant variation in different parts of the world. In India patients with respiratory symptoms are usually selected for viral testing. Various studies have shown that non-respiratory manifestations such as atrial fibrillation are also seen in COVID-19 [3]. Taste or olfactory disorders were noted in up to 53% of the cases in a small cohort from Italy [4]. Case series report gastrointestinal symptoms in 2-40% of patients [5,6]. About 80% of SARS-CoV-2 infections in ambulatory patients manifest as a mild respiratory illness and could usually be managed by outpatient care. About 15% of patients need inpatient care for moderate to severe pneumonia [7].

We aimed to describe epidemiological characteristics and symptoms of patients confirmed to have COVID-19 disease and to identify factors associated with the severity of the disease. We hope our study findings will inform the global community of the spectrum of symptoms of this novel coronavirus.

## Materials and methods

Study design and participants

This prospective cohort study was carried out from May 2020 to July 2020, at a tertiary care teaching hospital located in Central India and catering to both urban and rural populations. Study participants were all patients diagnosed and admitted with COVID-19, in a tertiary care hospital in central India from May 2020 to July 2020. Only laboratory-confirmed cases that were defined as positive based on the results of real-time reverse transcriptase-polymerase chain reaction (RT-PCR) assay of nasal and pharyngeal swab samples were included. The study was initiated following approval from the institutional ethics committee. Written informed consent was obtained from all the participants. A total of 150 patients who met the study criteria were included. During this period, all patients with RT-PCR test positive for COVID-19 with mild-to-moderate symptoms or even no symptoms were admitted to this hospital. Patients with severe symptoms (pulse oximeter oxygen saturation less than or equal to 80%) were not admitted because of the unavailability of a critical care unit in this newly developing hospital.

Data management and statistical analysis

A pre-tested semi-structured interview schedule was used for data collection. A team of doctors who had treated these patients extracted the recent exposure history, clinical symptoms, comorbidities, personal and past history from patients. All laboratory tests were performed according to treatment needs. Patients with COVID‐19 having severe illness were defined as having one of the following criteria: (a) respiratory distress with a respiratory rate more than or equal to 30/min or (b) pulse oximeter oxygen saturation less than or equal to 93% at rest. Clinical characteristics were compared between severe and non-severe cases. Since relevant data for the Indian population was not available at the beginning of the study, sample size was not estimated. Hence, it was decided to estimate power at the end of the study.

Statistical analyses were performed with SPSS version 24.0 (Chicago, IL: IBM Corp.). Continuous variables are described as median values and interquartile ranges (IQRs), and categorical variables are reported as numbers and percentages. Fisher exact test was used to determine any associations between two categorical variables.

## Results

We obtained data on the demographic characteristics, symptoms, and outcomes for 150 patients hospitalized from in our hospital May 2020 to July 2020. The demographic and clinical characteristics of the patients are shown in Table 1. The median age of the patient was 31.5 years (IQR, 17-45 years). Male patients accounted for 58% of all patients. A history of tobacco consumption was noted for 4.0% of the 150 patients. At the time of admission, 52.7% (79/150) patients were symptomatic while 47.3% (71/150) patients were asymptomatic. The symptoms reported by 79 patients are presented in Table 2.

**Table 1:** Demographic distribution and clinical characteristics of the study sample on admission

Sociodemographic characteristic	Categories	N (%)
Age	0-9 year	16 (10.7)
	10-19 year	31 (20.7)
	20-29 year	30 (20.0)
	30-39 year	22 (14.7)
	40-49 year	31 (20.7)
	50-59 year	12 (8.0)
	60 years and above	8 (5.3)
Gender	Female	63 (42.0)
	Male	87 (58.0)
Symptom status	Asymptomatic	71 (47.3)
	Symptomatic	79 (52.7)
Contact history with a known confirmed case	Positive contact history	141 (94.0)
Travel history	Positive travel history	10 (6.7)

**Table 2:** Distribution of symptoms in symptomatic COVID-19 patients COVID-19: coronavirus disease 2019

Symptom	N (%)
Fever	32 (40.5)
Sore throat	29 (36.7)
Cough	26 (32.9)
Rhinitis	15 (19.0)
Body ache	11 (13.9)
New loss of smell	10 (12.7)
Breathlessness	8 (10.1)
Headache	8 (10.1)
New loss of taste	5 (6.3)
Expectoration	1 (1.3)
Diarrhea	1 (1.3)
Nausea	1 (1.3)
Rash	1 (1.3)
Chills	1 (1.3)

The most common symptom of COVID-19 was fever, which was observed in 40.5% (32/79) of the patients. The other common symptoms were sore throat (36.7%, 29/79), cough (32.9%, 26/79), rhinitis (19.0%, 15/79), body ache (13.9%, 11/79), new loss of smell (12.7, 10/79), breathlessness (10.1%, 8/79), and headache (10.1%, 8/79). Less common symptoms were new loss of taste (6.3%, 5/79), expectoration, diarrhea, nausea, and chills (1.3%, 1/79). At least one comorbidity was reported in 20.0% (30/150) of the patients, with the most common comorbidity being hypertension (14.7%, 22/150) followed by diabetes (4.7%, 7/150) and ischemic heart disease (2%, 3/150) as shown in Table 3.

**Table 3:** Frequency/percentage of comorbid conditions associated with COVID-19 patients COVID-19: coronavirus disease 2019

Type of comorbidity	N (%)
Hypertension	22 (14.7)
Diabetes mellitus	7 (4.7)
Ischemic heart disease	3 (2.0)
Asthma	3 (2.0)
Acquired immunodeficiency syndrome	1 (0.7)
Sickle-cell anemia	1 (0.7)
Hypothyroidism	1 (0.7)

History of contact with a known confirmed case of COVID-19 within the last 14 days was present in 94% (141/150) patients. A history of traveling (intra-country) in recent 15 days was present in 6.7% (10/150) patients. The severe group included five (3.3%) patients while the non-severe group included 145 (96.7%) patients. Patients with severe disease were older than those with a non-severe disease by a median of 14 years. The presence of any coexisting illness was significantly higher among patients with severe disease than among those with non-severe disease (80% vs. 17.9%, p=0.012) which suggests a significant association of the presence of coexisting illness with the severity of COVID-19 as depicted in Table 4.

**Table 4:** Comparison between severe and non-severe COVID-19 disease group COVID-19: coronavirus disease 2019

	Number of patients	Median age in years	Comorbidity
Severe disease, N (%)	5 (3.3%)	50	4 (80%)
Non-severe disease, N (%)	145 (96.7%)	29	26 (17.9)
Total, N	150	31.5	30
p-Value	-	-	0.012

## Discussion

Understanding the clinical characteristics of COVID-19 in patients is very important for controlling the spread of COVID-19 and decision-making for epidemic control. In our study, we analyzed the symptoms of 150 patients with COVID‐19. Among them, 52.7% of patients were symptomatic while 47.3% of patients were asymptomatic, which was consistent with the previous report in which it was 48.2% and 51.8%, respectively [8]. A study by Rothe et al. indicates that a runny nose or sore throat can be isolated symptoms [9]. Testing strategies that exclude patients with few symptoms are likely to miss a substantial proportion of cases.

Available evidence indicates that up to 12% of transmission occurs before an index case develops symptoms [10,11]. This has important implications for the effectiveness of any testing strategy, contact tracing, and containment measures. To curtail active transmission of SARS-CoV-2, testing should be extended far beyond people who fit a narrow case definition and other populations currently considered at risk. Broad population screening for SARS-CoV-2 infections, isolation of confirmed cases through contact tracing and quarantine combined with social distancing, and large serological studies will be critical to slowing the spread of COVID-19.

In this study, the most common symptom of COVID-19 was fever, which was observed in 40.5% (32/79) of the patients, which was consistent with the previous report of Lechien et al. in which it was 45.4% [12]. In our study, hypertension and diabetes (14.7% and 4.7%, respectively) were the most common comorbidities. Similar findings were seen in the study of Zhang et al. in which it was 16.4% and 9.8%, respectively [13]. In this study, fever and cough were the most common symptoms. Similarly, an analysis of data from 4203 patients mostly from China identified fever, cough, and dyspnea (80.5%, 58.3%, and 23.8%, respectively) as the most common clinical symptoms [13].

Based on the current research, olfactory dysfunction has a high incidence rate in COVID-19 patients in some European and American countries, while it rarely occurs in Chinese patients [14,15]. Our study reported a new loss of smell sensation in 12.7% (11/79) patients while Vaira et al. reported olfactory dysfunction in 19.4% of patients, Yan et al. reported in 68%, and Lechein et al. reported olfactory dysfunction in 85.6% of patients [16-18]. More studies are required to find out reasons for variation in the occurrence of an olfactory disturbance in COVID-19 patients of different parts of the world.

In our study, approximately 6% of patients of COVID-19 had a loss of taste (ageusia). Lee et al. reported ageusia in 15.3% of patients [19]. Meini et al. reported gustatory dysfunction in 41% of patients [20]. Anosmia and ageusia seem to be part of important symptoms and clues for the diagnosis of COVID-19, particularly in the early stage of the disease. It should be remembered that non-specific symptoms such as headache, loss of taste, diarrhea, nausea, and rash could be the only sign with which to recognize a COVID-19 case. Awareness of such a non-specific presentation of COVID-19 patients is crucial during this pandemic period for preventing infectious spread through isolation and early initiation of COVID-19 targeted treatment.

In our study, the presence of any coexisting illness was significantly higher among patients with severe disease than among those with non-severe disease (80% vs. 17.9%, p=0.012) with estimated power at 80.6%. Guan et al. reported that multiple comorbidities are associated with the severity of COVID-19 disease progression. Many of the poorer outcomes for COVID-19 have been related to cardiovascular comorbid conditions [21]. Therefore, patients with comorbidities should take all necessary precautions to avoid getting infected with COVID-19, as they usually have the worst prognosis. Hence, there is a need for a global public health campaign to raise awareness, on reducing the burden of these comorbidity illnesses causing deaths in COVID-19-infected patients. Patients with severe disease were older than those with a non-severe disease by a median of 14 years. Hence old age can be considered as an independent risk factor for severe COVID-19 infection.

Our study has some notable limitations. First, only non-critical cases of COVID-19 were admitted to our hospital because of shortage of infrastructure and non-availability of intensive critical care unit. So our study cohort may represent the milder end of COVID-19. Second, we included symptoms and other parameters only on the day of admission and there was no follow-up. So we might have missed some symptoms that developed late during hospitalization. This is a modest-sized case series of patients admitted to the hospital; a collection of standardized data for a larger cohort would help to further define the clinical presentation, natural history, and risk factors. Further studies in outpatient, primary care, or community settings are needed to get a full picture of the spectrum of clinical severity. Despite these limitations, this study provides evidence on the main socio-demographic and clinical symptoms of confirmed cases of COVID-19.

## Conclusions

Approximately half of the COVID-19 patients in our study were asymptomatic. Fever and cough were the most common symptoms. The presence of any coexisting illness was significantly higher among patients with severe disease than among those with a non-severe disease. Patients with severe disease were older than those with a non-severe disease.

We documented the epidemiological characteristics and clinical symptoms of COVID-19 among patients from Central India. In our study, hypertension and diabetes were the most common comorbidities. Patients of advanced age with comorbidities were found to have more severe diseases. Further extensive studies with larger sample sizes from different parts of the world are required to gain deeper insights into COVID-19, which would lead to a better characterization of the course of this disease.
